# tModBase: deciphering the landscape of tRNA modifications and their dynamic changes from epitranscriptome data

**DOI:** 10.1093/nar/gkac1087

**Published:** 2022-11-21

**Authors:** Hao-Tian Lei, Zhang-Hao Wang, Bin Li, Yang Sun, Shi-Qiang Mei, Jian-Hua Yang, Liang-Hu Qu, Ling-Ling Zheng

**Affiliations:** MOE Key Laboratory of Gene Function and Regulation, State Key Laboratory of Biocontrol, School of Life Sciences, Sun Yat-sen University, Guangzhou 510275, P.R. China; Division of Biosciences, University College London, London WC1E 6BT, UK; MOE Key Laboratory of Gene Function and Regulation, State Key Laboratory of Biocontrol, School of Life Sciences, Sun Yat-sen University, Guangzhou 510275, P.R. China; MOE Key Laboratory of Gene Function and Regulation, State Key Laboratory of Biocontrol, School of Life Sciences, Sun Yat-sen University, Guangzhou 510275, P.R. China; MOE Key Laboratory of Gene Function and Regulation, State Key Laboratory of Biocontrol, School of Life Sciences, Sun Yat-sen University, Guangzhou 510275, P.R. China; MOE Key Laboratory of Gene Function and Regulation, State Key Laboratory of Biocontrol, School of Life Sciences, Sun Yat-sen University, Guangzhou 510275, P.R. China; MOE Key Laboratory of Gene Function and Regulation, State Key Laboratory of Biocontrol, School of Life Sciences, Sun Yat-sen University, Guangzhou 510275, P.R. China; MOE Key Laboratory of Gene Function and Regulation, State Key Laboratory of Biocontrol, School of Life Sciences, Sun Yat-sen University, Guangzhou 510275, P.R. China

## Abstract

tRNA molecules contain dense, abundant modifications that affect tRNA structure, stability, mRNA decoding and tsRNA formation. tRNA modifications and related enzymes are responsive to environmental cues and are associated with a range of physiological and pathological processes. However, there is a lack of resources that can be used to mine and analyse these dynamically changing tRNA modifications. In this study, we established tModBase (https://www.tmodbase.com/) for deciphering the landscape of tRNA modification profiles from epitranscriptome data. We analysed 103 datasets generated with second- and third-generation sequencing technologies and illustrated the misincorporation and termination signals of tRNA modification sites in ten species. We thus systematically demonstrate the modification profiles across different tissues/cell lines and summarize the characteristics of tRNA-associated human diseases. By integrating transcriptome data from 32 cancers, we developed novel tools for analysing the relationships between tRNA modifications and RNA modification enzymes, the expression of 1442 tRNA-derived small RNAs (tsRNAs), and 654 DNA variations. Our database will provide new insights into the features of tRNA modifications and the biological pathways in which they participate.

## INTRODUCTION

Transfer RNA (tRNA) is a key molecule in the process of decoding genetic information. tRNA molecules contain dense, abundant chemical modifications and may represent a record of the ancient RNA world (1–5). Approximately 400 unique tRNA molecules can potentially be produced in human cells (6,7). On average, each tRNA carries 13 modification sites (8). Moreover, recent studies have shown that tRNA modifications are highly dynamic and show a wide range of responses to cellular metabolite levels and environmental stresses (9–16). Furthermore, these changes in tRNA modifications affect tRNA structure, codon recognition, and tRNA-derived small RNA (tsRNA) formation (17–24). A growing number of studies have found that alterations in tRNA modifications can cause serious diseases, referred to as RNA modopathies (25). These studies have led a new chapter in tRNA modification research, and future work will surely reveal the mechanisms of dynamic tRNA modifications.

There are several databases that collect tRNAs or modifications thereof. GtRNAdb (26) is the most comprehensive tRNA database, but it mainly collects the sequences and structures of tRNAs. Similarly, the T-psi-C database (27) focuses on the presentation of tRNA sequences and 3D structures. The Modomics database (28) provides comprehensive information concerning the chemical structures of modified ribonucleosides and their biosynthetic pathways. However, this database mainly includes early published results for individual tRNAs and lacks large-scale tRNA epitranscriptome data. RMBase is a database developed by our team based on high-throughput sequencing data to analyse RNA modifications (29). However, it collects data from genome-wide RNA modification detection techniques, mainly including m6A modifications on mRNAs. These databases are either relatively old or do not specifically focus on tRNA modifications, making it difficult to reveal the full landscape of the tRNA epitranscriptome.

Several epitranscriptomic technologies have been developed in recent years, which use special library construction strategies and identify tRNA modifications according to reverse transcription (RT) stop and misincorporation signatures (7,30–33). The most recent third-generation single-molecule sequencing strategy allows the direct detection of modifications on RNA molecules (34–38). However, current research on tRNA modopathies still faces many difficulties. First, each tRNA sequencing technology identifies tRNA modifications according to a different strategy. For example, QuantM-tRNA-Seq identifies modification sites based on the stop signal of tRNA fragments. mim-tRNA-seq (7) uses the RT misincorporation signature to identify tRNA modifications, while ARM-Seq (39) and DM-tRNA-seq (40) require the comparison of data obtained before and after AlkB treatment to demethylate common modifications in tRNAs. RBS-seq (41), developed based on bisulphite sequencing (42–45), utilizes chemical treatment to specifically identify m1A, Ψ and m5C. AQRNA-seq uses exonucleases to remove excess adapters to measure tRNA abundance and modification changes (31). The most recent development of Nanopore tRNA sequencing technology (34) allows the direct sequencing of tRNA molecules without reverse transcription steps, but it also produces a large number of miscalls. The differences in these sequencing technologies result in data including modification sites exhibiting completely different characteristics, making it difficult to compare data with each other. Second, the sequences of different tRNA isotypes are very similar, meaning that standard mapping strategies cannot precisely annotate the type of modifications at each site of each tRNA molecule. This can make it very difficult to identify the dynamic changes in these modifications at later stages. Finally, tRNA modifications are influenced by structural changes in tRNAs and differences in the levels of modification enzymes, and they determine the recognition of codons and the processing of tsRNAs (18–23). Current tRNA databases cannot store and display these complex factors simultaneously, thus limiting the subsequent resolution of their mechanisms and functions.

In summary, the detection of tRNA modifications requires specialized library construction strategies and bioinformatic analysis methods to ensure accuracy and high resolution. Based on the above research needs and problems, this study developed tModBase, a specialized resource for deciphering the landscape of tRNA modification profiles from epitranscriptome data.

## MATERIALS AND METHODS

### Data acquisition and download

For *Homo sapiens* (GRCh38/hg38), *Mus musculus* (GRCm39/mm39), *Saccharomyces cerevisiae* (S288c), *Schizosaccharomyces pombe* (972h-), *Drosophila melanogaster* (BDGP Rel. 6/dm6), *Arabidopsis thaliana* (TAIR10), *Oryza sativa japonica* group (Japanese rice IRGSP-1.0), *Medicago truncatula* A17 (MedtrA17_4.0), *Mycobacterium bovis* BCG (str. Pasteur 1173P2) and *Escherichia coli* (str. K-12 substr. MG1655), the mature tRNA sequences from GtRNAdb (26) were adapted, and identical sequences were merged at the isodecoder level. We used BLASTN to best match the dot-bracket structure and canonical structure from tRNAdb for each sequence in the tRNA reference library. For species with mitochondrial tRNA, we obtained mitochondrial tRNA reference sequences, dot-bracket structures and canonical structures from mitotRNAdb (46) or tRNAdb (46). The above steps are performed using the Python script.

The raw sequencing data were obtained from SRA files obtained with various sequencing technologies in the Gene Expression Omnibus (GEO) (47) database. The RNA-seq transcriptome profiling data of normal tumour samples from 32 cancer types were obtained from the TCGA database, and FPKM values were calculated using counts to represent the gene expression levels of the modification enzyme. RPM values were calculated to represent the expression levels of tRNA molecules. In addition, human (hg38) SNP data were obtained from the dbSNP database (48).

### Annotation of known tRNA modifications and modification enzymes

Based on the available information on modifications for each species from the Modomics database modification were manually counted, and BLASTN (49) was used to align the modified sequences provided by Modomics with the compiled isodecoder sequence set. Modifications matching the tRNA molecules were screened from RMBase as complementary data. For human mitochondrial modifications, we collected more comprehensive annotation information from the literature (50).

### tRNA quantification and identification of modification signals in epitranscriptome sequencing data

We collected sequence read data from 103 datasets of 11 high-throughput sequencing (HTS) technologies (Supplementary Table S1). These raw sequencing reads were subjected to quality control and sequence alignment, and the alignment results were quantified with the custom Python scripts.

- **DM-tRNA-seq**

Before mapping, we used Trim_Galore! v0.6.7 for the quality control of all sequencing datasets from DM-tRNA-seq (40) with the following parameters:

-q 20 –phred33 –stringency 5 –length 15 -e 0.1

followed by alignment to the reference library using Bowtie2 (51) with the parameters:

D 20 -R 3 -N 1 -i S,1,0.50.

- **ARM-seq**

We used the quality control tool Trim_Galore! v0.6.7 to trim reads with the following parameters:

-q 20 –phred33 –stringency 5 –length 15 -e 0.1

Followed by alignment to the reference library using Bowtie2 (51) with the following parameters:

–quiet –min-score G,1,8 –local -D 20 -R 3 -N 1 -L 10 -i S,1,0.5.

Multiple mapping reads were filtered out with the custom Python scripts.

- **YAMAT-seq**

Since the original article for YAMAT-seq (30) did not specify quality control tools or parameters, we used Trim_Galore! v0.6.7 for the quality control of YAMAT-seq reads with the following parameters:

-q 20 –phred33 –stringency 5 -e 0.1

Since the maintenance of SHRiMP2, used in the YAMAT-seq article, had stopped, we used Bowtie2 (51) instead with the following parameters:

–quiet –min-score G,1,8 –local -D 20 -R 3 -N 1 -L 10 -i S,1,0.5.

- **hydro-tRNA-seq**

We trimmed the hydro-tRNA-seq reads using Cutadapt v4.1 (52), and the 3′ adapter sequences of 4 replicates were as follows:

TCGTATGCCGTCCTCTGTTTG; TCACTTCGTGTCGTTCTGTGTGT; TCACTTCGTGTGCCGTCCTCTGTGTGTG; TCTAGTCGTATGCCGTCTTCTCTCTGTG

For sequence alignment, the default parameters of BWA-MEM were used.

- **QuantM-tRNA-seq**

Commands for quality control and alignment were provided in the QuantM-tRNA-seq reference:

cutadapt –cut 2 -a CCAGTATCCAGTTGGAATT -g TCCAACTGGATACTGGN -e 0.2

bowtie2 –quiet –min-score G,1,8 –local -D 20 -R 3 -N 1 -L 10 -i S,1,0.5

- **mim-tRNA-seq**

We used the pipeline provided by mim-tRNA-seq (7) for bioinformatics analysis and processed the results of the pipeline into a unified format through the custom script.

- **AQRNA-seq**

In AQRNA-seq, fastxtoolkit was used for read quality control, but the adapter sequences were not given. Hence, we used Trim_Galore! v0.6.7 instead with the following parameters:

-q 20 –phred33 –stringency 5 –length 10 -e 0.1

We used BLAST for sequence alignment, and the parameters were as follows:

blast -perc identity 90 -word_size 9 -dust no -soft_masking false

- **BS-seq**

We use the default parameters of Bismark (53) throughout the analysis of BS-seq data.

- **Pseudo-seq**

The following command was used to perform quality control and sequence alignment as the Pseudo-seq article provided:

cutadapt -a ADAPTER_SEQ –overlap 3 –minimum-length 18 -o trimmed_reads.fastq.gz input_reads.fastq.gz.

bowtie2 –quiet –min-score G,1,8 –local -D 20 -R 3 -N 1 -L 10 -i S,1,0.5

The Pseudo-seq peak value was calculated for the paired + CMC/-CMC samples with the following formula:

$$\begin{equation*}{\rm{peak\ }} = {\rm{\ ws}}\frac{{{r}^ + - {r}^ - }}{{w{r}^ + + w{r}^ - }}\end{equation*}$$

Here, ws is the window size, which is set to 10 because the reference tRNA sequences are small. In addition, r^+^ and r^−^ are the numbers of reads terminating at the 3′ ends of the + CMC sample and -CMC sample, respectively. Moreover, wr^+^ and wr^−^ are the numbers of all reads terminating within the windows for + CMC samples and -CMC samples, respectively. These calculations can be performed with the custom script.

- **m1A-seq**

The supplementary file of the m1A-MAP article provided results including mismatch information at various positions in all the tRNA sequences, and we sorted the results into a unified format with the custom script.

- **Nanopore sequencing**

After obtaining the original data (fast5 format) from Thomas *et al.* (34), we performed base calling using Guppy, which is recommended in the NanoProcess portion of the Master of Pores process. Then, we replaced U in the fastq format file with T. The obtained results were aligned with the reference tRNA sequences provided by the original article using Bowtie2 (51), filtered according to MAPQ scores greater than or equal to 1. Finally, the custom Python script was used to calculate the counts.

### Differential expression analysis of tRNA and RNA modification enzymes

We collected all RNA-modifying enzymes responsible for tRNA modification from databases (28,29) and the literatures (54–56) and classified them into deamination, methylation, pseudouridylation, oxidation, hydroxylation, thiolation, acetylation, dihydrouridine modification, taurine modification, queuine modification, guanylylation and alkylation enzymes, for a total of 12 categories. In addition, we divided tRNA modification enzymes into two types, writers and erasers, according to their modification functions. To study the changes in modification enzymes in cancers, we downloaded data from >10 000 samples from 32 cancers available in the TCGA database (57). FPKM values were used to evaluate the expression of all genes based on the transcriptome sequencing data, and the expression levels of various modification enzymes were extracted from these data.

To compare the differences in tRNA expression between tumour and normal tissues, we first downloaded raw small RNA sequencing data from the TCGA database and then mapped them on mature tRNA sequences to quantify the tRNA expression levels in each sample. The results were next normalized using the expression levels of the modification enzymes with the following formula:

$$\begin{equation*}NormtRN{A}_i\ = \ \frac{{tRNAex{p}_i}}{{\mathop \sum \nolimits_{j\ = \ 1}^{{n}_i} RMPex{p}_j}}\end{equation*}$$

where, for a specific tRNA molecule *i*, the normalized value is the expression level of the tRNA divided by the sum of the expression levels of the modification enzymes (RNA modification Protein, RMP) corresponding to all modification sites (from *j* to *n*_*i*_) on that tRNA.

### tsRNA level quantification and relationship analysis of modification sites

We previously established the tsRFinder tool for identifying tsRNA molecules from all small RNA samples in the TCGA database. Subsequently, we annotated the locations where the tsRNA molecules were generated on the tRNAs in detail in the identification results. The annotation results intersected with the modification sites on the tRNA molecules that we collected. Notably, studies have confirmed that the modification of tRNA has little effect on the identification of tsRNAs based on the TCGA library construction method (58).

### Analysis of the relationship between RNA modification and DNA variation sites

To analyse the relationships between tRNA modification sites and known DNA variations, we downloaded the sites of known human DNA variations, including SNVs, insertions, deletions, and indels, from the dbSNP database. Then, the bedintersect tool (59) was used to identify the intersection between various DNA variants and all positions of tRNA modification sites in the reference tRNA sequences.

## RESULTS

### Dynamic interaction of tRNA modification positions and corresponding modification enzymes

**Figure 1.**
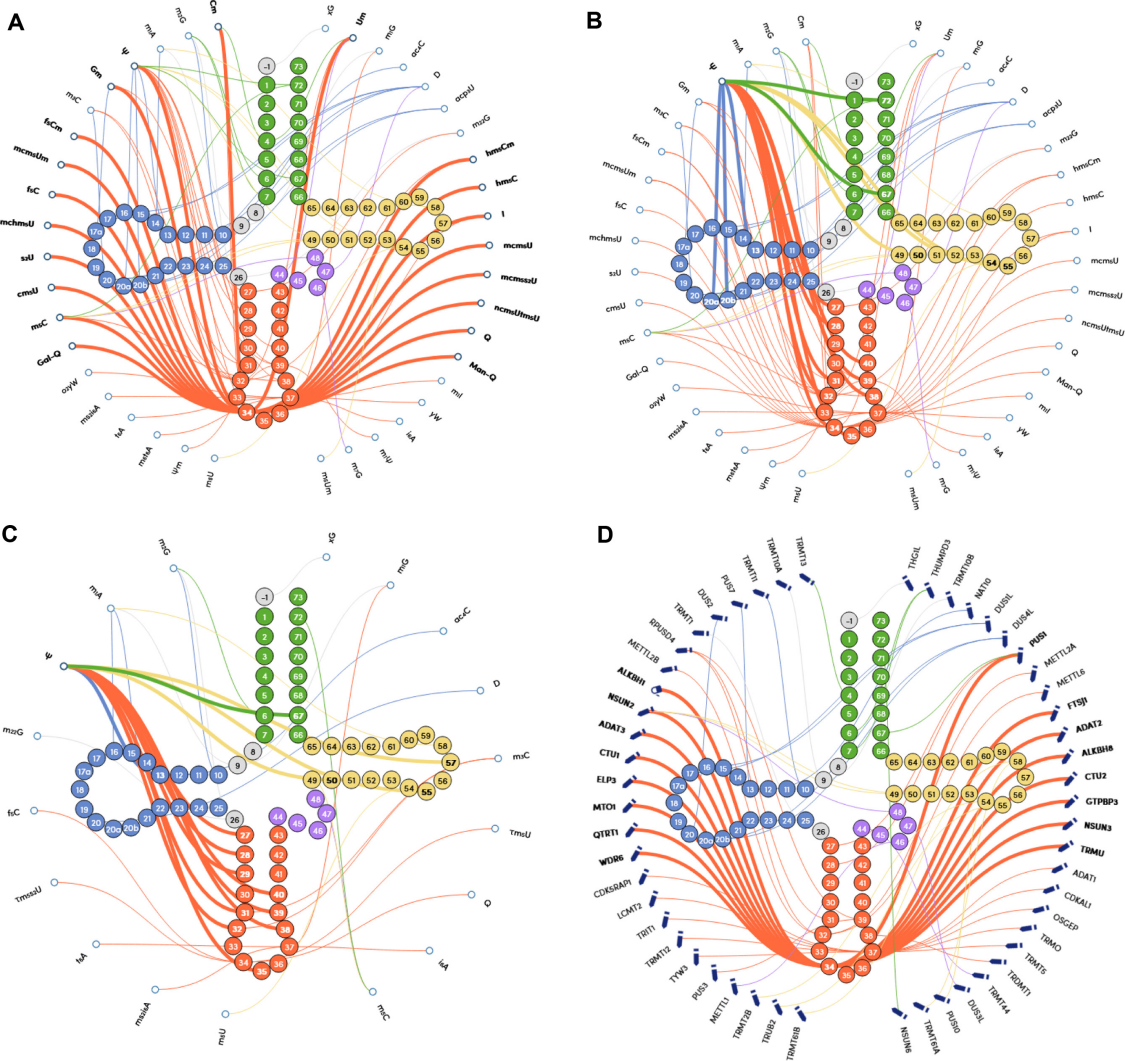
Dynamic interaction diagram of the modifications and modification enzyme types corresponding to each site in the tRNA consensus structure. (**A**) Modifications corresponding to each site in cytoplasmic tRNA, where the modification types at position 34 are highlighted. The type of modification corresponding to each site in cytoplasmic (**B**) and mitochondrial (**C**) tRNA molecules, with the modified sites affected by pseudouridine highlighted. (**D**) Map of possible tRNA sites of RNA-modifying enzymes, with the modification enzymes that affect the modification at position 34 of tRNA highlighted. The pencil icon represents an enzyme with writer function, and the eraser icon represents an enzyme with eraser function.

A graph of the interaction between tRNA structure and modification is available on the tModBase home page (Figure 1). Users can intuitively check the modification type of each site in the consensus sequence of human cytoplasmic and mitochondrial tRNAs. For example, in human cytoplasmic tRNAs, position 34 is the site with the most abundant modification types, including a total of 20 modification types (Figure 1A). Additionally, this site corresponds to the wobble position of the mRNA codon, which affects the accuracy of codon recognition and the fidelity of translation. It can also be seen from the figure that whether the tRNA is cytoplasmic or mitochondrial, the most commonly affected modification site is pseudouridine (Ψ), and the bases with this modification are mainly distributed in the anticodon arm structure of the tRNA clover (Figure 1B, C).

Different types of tRNA modifications are mediated by a variety of RNA modification enzymes (RNA modification proteins, RMPs). tModBase maps the modification types at each site to the corresponding modification enzymes and divides the modification enzymes into writers and erasers. For example, tRNA position 34 may be affected by 15 different writer enzymes (NSUN2, NSUN3, ADAT2, ADAT3, CTU1, CTU2, ELP3, MTO1, QTRT1, WDR6, PUS1, FTSJ1, ALKBH8, GTPBP3 and TRMU) and one eraser enzyme (ALKBH1) (Figure 1D). Users can easily search for modification enzymes based on the modification of interest and may search for the type and site of tRNA modification affected by a specific enzyme. In addition to the interactive diagram on the home page, users can access and filter more detailed information through three pages: tRNA, Modification, and Enzyme.

In conclusion, the currently known tRNA modification sites in humans and mice are well annotated in tModBase. However, we found that these annotated tRNAs represented only a small fraction of the total tRNA isotypes (Table 1). For example, the human genome contains a total of 432 tRNA loci, encoding 283 different tRNA isotypes, only 54 of which have well-annotated modification sites, accounting for 19% of all isotypes. Thus, the question arises of which sites on the remaining more than 80% of tRNA molecules are likely to contain annotations? We next mine through epitranscriptomic data.

**Table 1.** List of the ratios of annotated modified tRNA molecules to the total number of tRNAs

Species	# tRNA isotypes	# tRNA isotypes with annotated modification site	Percentage of known modified tRNAs (%)	# tRNA molecules with sequencing signals identified in epitranscriptomic data	Percentage of potential modified tRNAs identified by tModBase (%)
*Homo sapiens*	283	54	19	274	97
*Mus musculus*	240	12	5	201	84
*Saccharomyces cerevisiae*	81	54	67	77	95
*Escherichia coli*	49	38	76	43	88

### Mining tRNA modification signals from epitranscriptomic data

**Figure 2.**
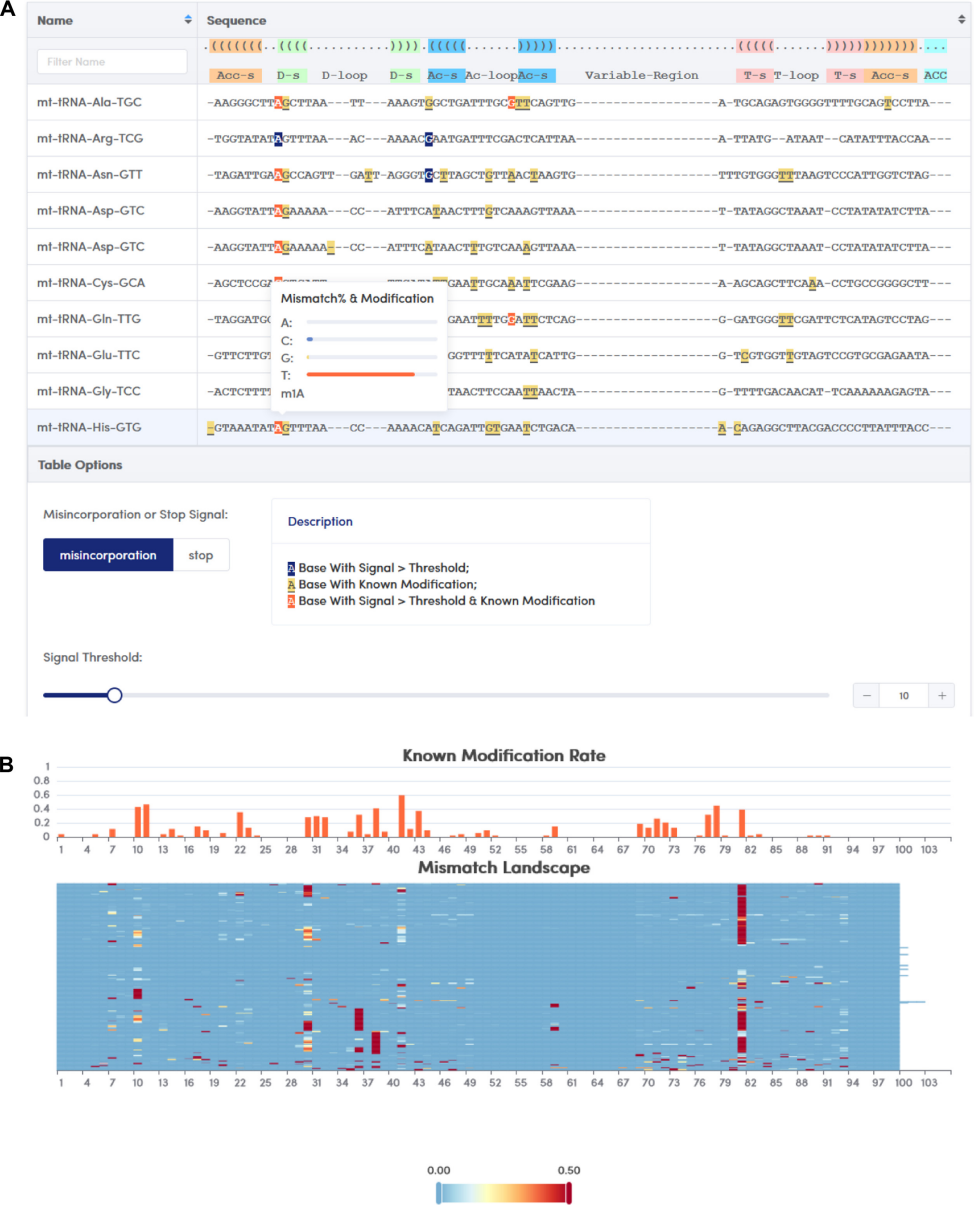
Distribution of tRNA epitranscriptome sequencing signals displayed in tModBase. (**A**) Each row in the table represents the misincorporation signal detected in a tRNA isotype sequence. (**B**) The x-axis in the histogram represents the tRNA site in the consensus structure, and the y-axis represents the number of known modification types at that site. Each row of the heatmap represents a tRNA isotype, each column represents a site in the consensus structure of the tRNA, and the colour indicates the intensity of the misincorporation signal, such as the percentage of mismatches.

In recent years, a series of tRNA epitranscriptome sequencing technologies have been developed to obtain high-throughput tRNA modification signals. We collected and analysed 103 high-throughput sequencing datasets based on 11 library construction strategies for tRNA quantification and the identification of modification types from species including *Homo sapiens*, *Mus musculus*, *Saccharomyces cerevisiae*, *Schizosaccharomyces pombe*, *Drosophila melanogaster*, *Arabidopsis thaliana*, *Oryza sativa*, *Medicago truncatula*, *Solanum tuberosum*, *Mycobacterium tuberculosis variant bovis BCG* and *Escherichia coli* (Supplementary Table S1). We developed corresponding analysis workflows for different library construction strategies (see Methods), and finally obtained the distribution of the modification signals (misincorporation or termination) in each tRNA molecule (Figure 2A). tModBase shows the overlapping relationships between the sites of different modification signals and the known modification sites in the molecule in detail. For example, according to the annotation, there is an m1A modification at the 9th site of mt-tRNA-His-GTG. Using mim-tRNA-seq technology (7), it can be observed that this site shows a strong A to T mismatch signal, which is determined by the properties of TGIRT RTase (60–62).

To better illustrate the common features of the distribution of modification signals on multiple tRNAs, we generated a heatmap of the distribution of modification signals of multiple tRNAs, corresponding to known modification sites, based on which the proportion and extent of the signal distribution on various tRNA molecules could be clearly displayed (Figure 2B).

### Comparison of tRNA modification profiles between different tissues/cell lines

**Figure 3.**
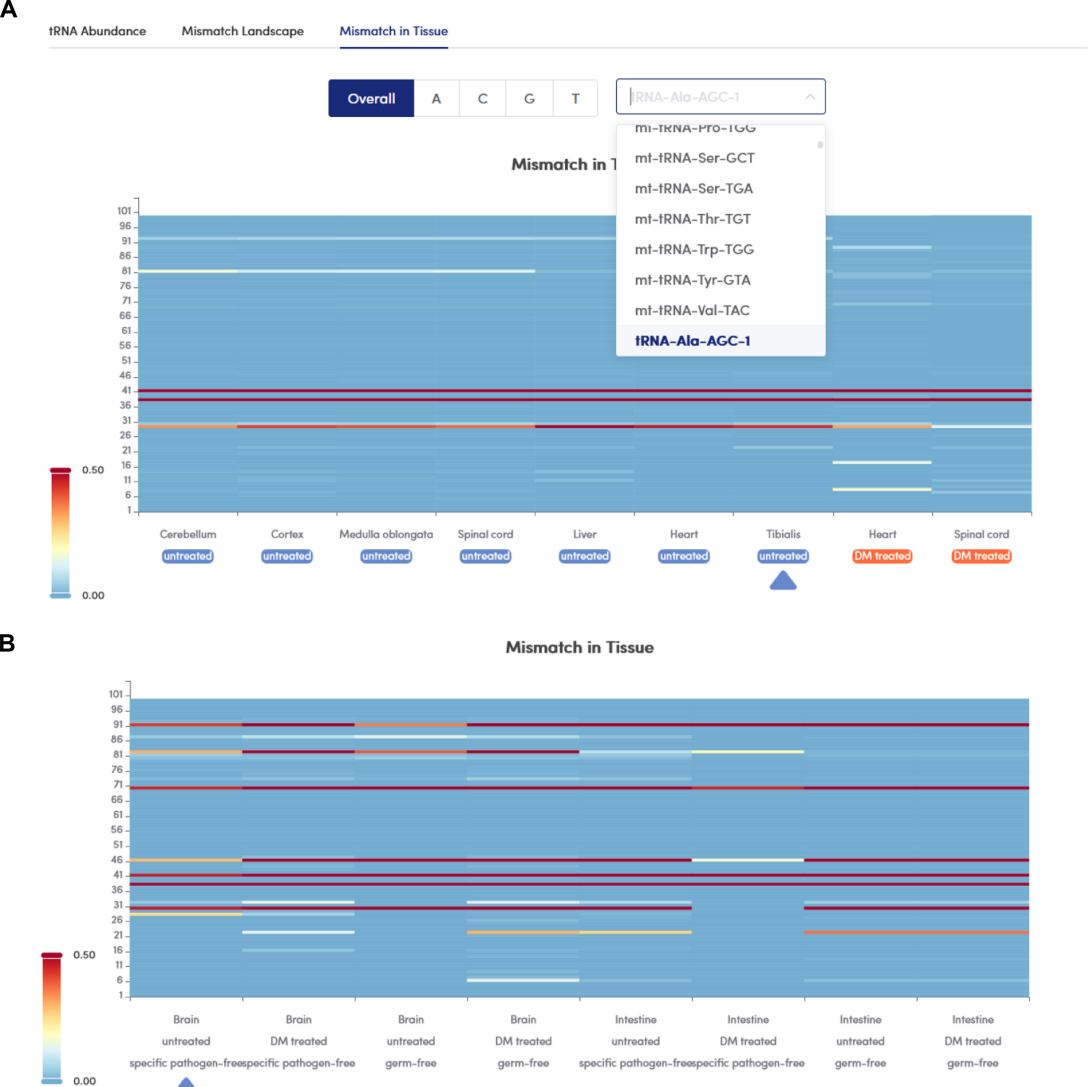
Modification profiles displayed in tModBase. Each row represents a condition: (**A**) different tissues of mouse and (**B**) the presence or absence of microorganisms, and each column represents a site on the tRNA molecule. The colour indicates the strength of the misincorporation signal, such as the percentage of mismatches.

tModBase provides modification signal profiles recorded under various conditions, including profiles from three human cell lines (HEK293, K562 and iPSC) (Figure 3A), nine types of mouse tissues (heart, liver, cerebellum, cortex, medulla oblongata, spinal cord, tibialis, brain, and intestine) and different time points of T-cell activation in mice (naïve, 20 h after activation, 48 h after activation, 72 h after activation). In addition, tModBase provides the results of comparisons between the modification profiles of mouse tissues with or without microbial or virus infection (Figure 3B). Users can select tRNAs of interest to view the changes in the modification signals at various sites under different conditions.

**Figure 4.**
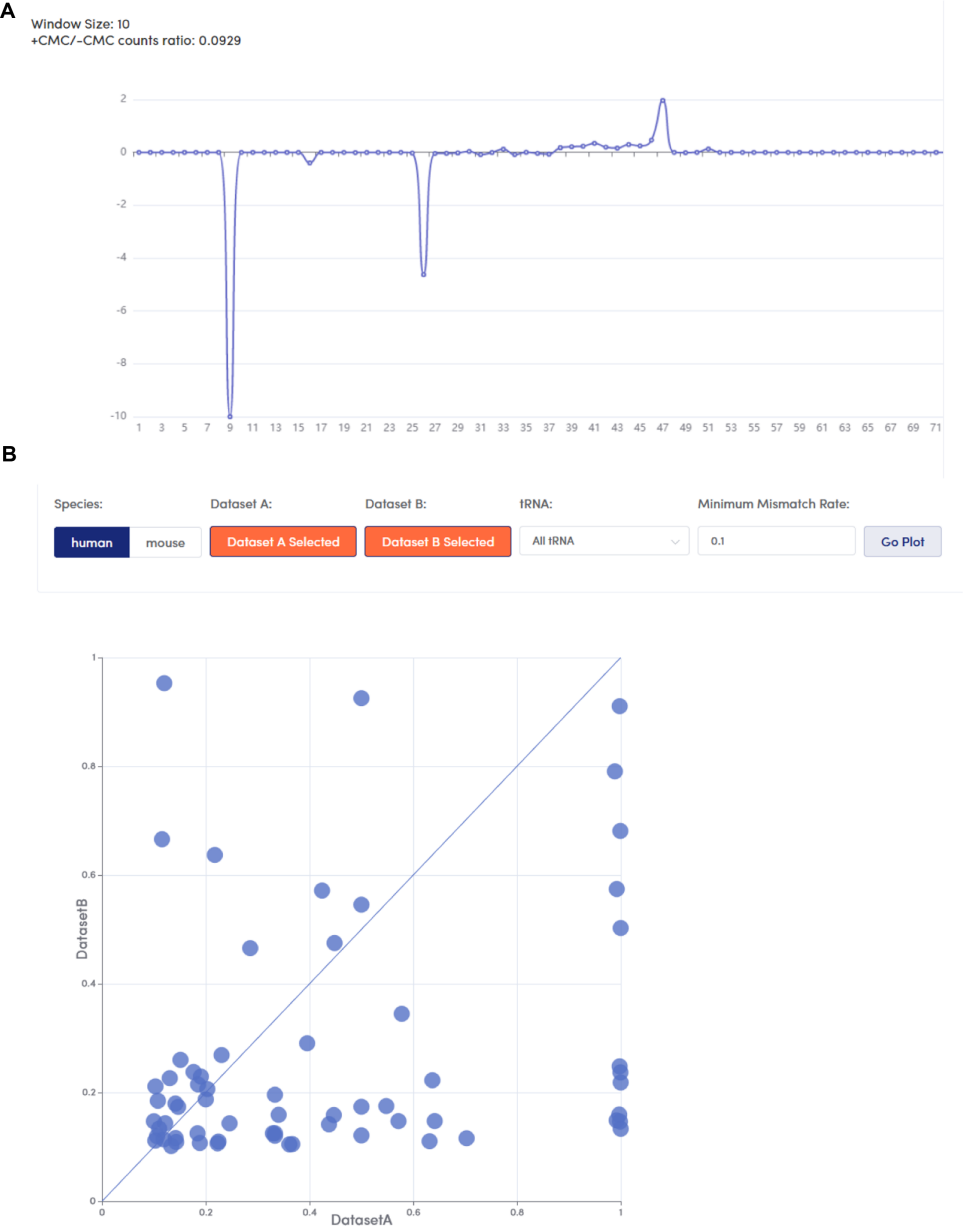
Comparison of modification profiles between different sequencing sets. (**A**) Distribution of misincorporation signal ratios between chemically treated and control groups. The x-axis represents each position of a tRNA molecule, and the y-axis represents the ratio of the misincorporation signal between chemically treated and control samples. (**B**) Scatter plot comparing the two datasets. The x- and y-axes represent the full range of misincorporation signal values in the two datasets selected by the user, with each point representing a site on a tRNA molecule.

Some sequencing methods (such as Pseudo-seq) require comparisons of datasets before and after chemical treatments (such as carbodiimide and CMC treatment). As a result, we calculated the rates of changes in the sequencing signals before and after treatment at each tRNA locus (Figure 4A). In addition, tModBase provides an analysis tool, Mod2Compare, for comparing any two epitranscriptome sequencing signals (Figure 4B). In Mod2Compare, the user can select any two datasets for comparison to obtain a downloadable table showing the misincorporation signal values detected in a common or unique part of the two datasets. In addition, mod2Compare provides an upload function, and the user can not only compare any two datasets contained in tModBase but can also upload a new dataset. With the two functions above, users can employ the first function to download the intersection or difference between any two datasets and then use the second function to reupload the results to a new dataset and compare it with a third dataset. Even more custom permutations can be performed by the user in a comparative manner.

### Association between tRNA modification and tsRNA biogenesis

tRNA modifications affect the structure of the tRNA and the degree of resistance to nucleases at specific sites, which in turn triggers tsRNA processing (63). We summarized a list of the relationships between tRNA modifications and tsRNA processing positions reported in the literature, which lists tsRNA types, modification sites, modification enzymes, and the corresponding mechanisms of action. Our previous study revealed that tsRNAs are abnormally expressed in a variety of cancers, which is associated with patient survival and prognosis (64). To further clarify the relationship between tsRNA occurrence and tRNA modifications, we analysed the biogenesis sites of tsRNAs in 32 cancers and mapped them to known tRNA modification sites. Since modification changes are affected by modification enzymes, we also displayed the changes in various modification enzymes in 32 cancers on the Enzyme page. Additionally, we provide a graph comparing tRNA expression levels between tumour and normal tissues on the tRNA page. Researchers can screen modification enzymes and tsRNA molecules that undergo synergistic changes in tModBase, and then design experiments to deeply explore the mechanisms of abnormal tsRNA levels in cancer.

### Diseases caused by tRNA modification

Disturbances in tRNA modification can cause serious diseases. However, the types of diseases identified thus far are still very limited. The relationships between reported tRNA modifications and four major disease types (mitochondrial disease, neurological disorder, cancer, and diabetes) are summarized in tModBase. The Mod2Disease page details disease names, affected genes, types of RNA modifications, and tRNA molecules. On this basis, we annotated whether the phenotype is caused by the influence of tRNA modification enzymes or the modifications themselves in detail and briefly summarized the corresponding pathogenic mechanisms (Supplementary Table S2). In addition, we analysed the relationships between tRNA modification sites and DNA variation sites. The tRNA molecules, modification sites, and modification types and DNA variation sites and variation types are annotated in detail. In the future, additional diseases caused by tRNA modification changes can be identified based on tModBase, and their pathogenic mechanisms can be further analysed.

## DISCUSSION AND CONCLUSIONS

**Figure 5.**
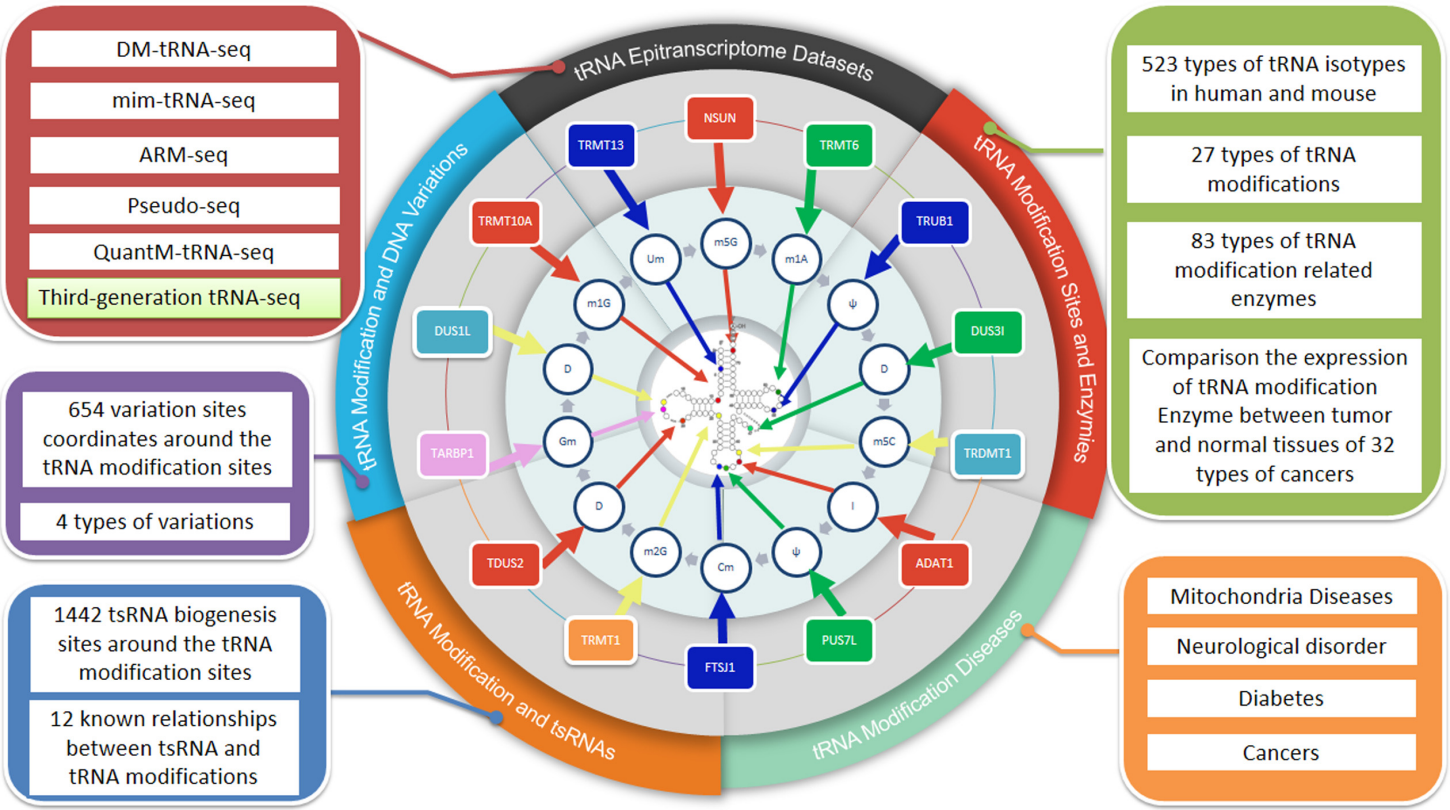
Major contents of tModBase.

Unlike mRNA modifications, modifications on tRNA molecules are abundant and densely distributed. Traditional biochemical methods can identify the types of modifications present on tRNA molecules but cannot perform large-scale batch detection, let alone distinguish multiple tRNA isotypes. In recent years, a series of high-resolution experimental detection methods and bioinformatics analysis procedures focused on tRNA modifications and quantification have been developed. This study integrates and analyses currently reported tRNA epitranscriptome sequencing datasets and demonstrates the misincorporation and termination signals generated by these sequencing technologies in detail (Figure 5). Additional tRNA molecules likely to contain modifications were identified. Based on the identified misincorporation signals, users can design experiments to further discover new tRNA-modifying molecules and even rare types of modifications. To avoid the problem of false-positives caused by the use of a single dataset, tModBase provides the Mod2Compare tool to compare the differences in the identification signals of the two datasets. It is recommended that researchers use the result obtained from the intersection of multiple datasets as a more credible modification signal for validation.

Many modifications can cause misincorporation or termination in the RT process, causing the site to generate a detectable signal suggesting that the site is a possible modification site. However, relying on such a signal alone may produce a large number of false-positives, as unmodified bases may also cause misincorporation or termination during the RT process. This is one of the issues that tModBase is trying to address, that is, to provide cross-validation results for multiple datasets to increase accuracy. In fact, there may be multiple reasons for misincorporation or termination, including the library construction method, experimental technique, base type, modification type, tRNA structure, RT enzyme characteristics, or sequencing errors involved. To reflect this information comprehensively, we built a summary table on the position page that shows how many datasets contained a signal at a site, the base type at the site, whether previous studies reported the presence of modifications at the site, the reverse transcriptase used for sequencing, etc. Users can determine the confidence of a modification site based on these features. In the future, researchers can train machine learning models to make more accurate predictions of tRNA modification sites based on the above information provided by tModBase as features.

In addition, many modification types can lead to incomplete reverse transcription of tRNA and, thus, the lose of this part of the signal, which is also a very important issue. Fortunately, the tRNA sequencing technology collected in tModBase has been designed to address this issue during the library construction process. The basic idea includes three key points: (i) A 3′ adapter is ligated to tRNA 3′ ends. The adapter-ligated tRNA pools are used as templates for primer-dependent cDNA synthesis by TGIRT. (ii) The cDNA is circularized with CircLigase to provide a template for library construction by PCR. (iii) The previously ligated adapter is cleaved using a restriction endonuclease, thus linearizing the circularized RNA again while ensuring that both ends of the target are successfully ligated with the adapter sequences. Hence, the truncated cDNA resulting from the RT stop can also be detected in these libraries. We have summarized a table of the datasets used in tmodBase, describing the library construction strategy for the sequencing data and the reverse transcriptase (RTase) that is used. In the future, researchers can train machine learning models to make more accurate predictions of tRNA modification sites based on the above information provided by tModBase as features.

Using tModBase, researchers can intuitively observe the signal preferences at known modification sites and correlate them with the corresponding modification enzyme signatures. For example, TGIRT, a reverse transcriptase, can cause an RT misincorporation signal of A to T or G at m1A (determined by the structural properties of the enzyme itself) (62). By summarizing the properties of different reverse transcriptases, researchers can further apply them for the identification of tRNA modification types.

tRNA modifications change dynamically under different conditions, and earlier studies have detected the tRNA modification changes during cellular changes under stress conditions (imposed by temperature, chemical reagent treatment, etc.) using LC–MS/MS techniques (Supplementary Table S3) (9–11,13–15,65,66). Recent studies using HTS techniques have identified specific tRNA modifications in human and mouse cells that are significantly altered under pathogenic infection (67,68) and during T cell activation (32). This evidence implies that dynamic changes in tRNA modifications may be a general response of cells to stress conditions; however, the modifications that can be detected by LC–MS/MS methods cannot clarify the specific tRNA isotypes, and the data obtained from HTS are currently far from sufficient. tModBase will be updated continuously to collect and demonstrate the dynamic changes in tRNA modifications. In the future, we will continue to collect more dynamic modification data generated by HTS and LC–MS/MS methods and add them to tModBase. In addition, we provide a user-submitted data function that allows researchers to upload these data into tModBase if relevant work is published, and we believe that having such a dedicated resource will help advance the field.

The identification and quantification of tRNA modifications are ultimately aimed at deciphering their mechanisms and functions, but research in this area is just emerging (69). In addition to collecting published reports, tModBase also provides tools such as Enzyme and Mod2tsRNA to correlate tRNA modification with changes in modification enzyme and tsRNA. In the future, researchers can integrate and analyse multiple factors to establish a regulatory network of tRNA modifications, RNA modification enzymes, and tsRNA levels to understand the molecular mechanisms that affect cell function and fate.

Compared with other tRNA and RNA modification databases focused mainly on tRNA sequences or modifications of other RNA molecules, tModBase has some distinct, important advantages (Figure 5). The advances in tModBase are as follows: (i) tModBase records 27 types of modifications, including m7G, t6A, Ψ and m1A, at each site in human and mouse tRNA molecules. (ii) We collected 103 datasets from 11 types of high-throughput tRNA sequencing technologies (Supplementary Table S1) and customized the data according to the applied library construction strategies. The signal of each sequencing technology and its dynamics under different conditions are demonstrated. In addition, tModBase provides tools for cross-sectional comparison between different sequencing results. (iii) tModBase matches the modification at each tRNA site to the expression of the enzyme that mediates that modification and shows the dynamics of these modification enzymes in 32 types of cancers. (iv) tModBase shows the overlap of tRNA modification sites with DNA variations, including SNVs, insertions, and deletions. Users can explore the variation sites corresponding to the modification sites in any tRNA and the associated diseases. (v) tModBase demonstrates the relationship between tRNA modification sites and the production positions of tRNA-derived small RNAs (tsRNAs). We provide the Mod2tsRNA tool to analyse the expression levels of tsRNA near tRNA modification sites, which helped to reveal the relationships between tRNA modifications and tsRNA production mechanisms. In addition, tModBase demonstrates the relationships between known tRNA modification sites and tsRNA processing from the published literature. (vi) tModBase includes four types of human diseases caused by aberrant tRNA modifications, including mitochondrial diseases, neurological disorders, cancers, and diabetes, and their associated tRNA molecules, modification types, and modification enzymes. (vii) The analysis results and display charts of all high-throughput sequencing data in tModBase are available for download so that users can conduct in-depth analysis and research based on tModBase.

## DATA AVAILABILITY

tModBase is freely available at https://www.tmodbase.com/.

## Supplementary Material

gkac1087_Supplemental_Files
